# Hyperprogression on immunotherapy with complete response to chemotherapy in a NSCLC patient with high PD-L1 and STK11: A case report

**DOI:** 10.1097/MD.0000000000022323

**Published:** 2020-11-13

**Authors:** Jeremy Fricke, Isa Mambetsariev, Rebecca Pharaon, Shanmuga Subbiah, Swapnil Rajurkar, Ravi Salgia

**Affiliations:** Department of Medical Oncology and Therapeutics Research, City of Hope National Medical Center, Duarte, CA.

**Keywords:** hyperprogressive disease, immune checkpoint inhibitors, nonsmall cell lung cancer, PD-L1, pembrolizumab

## Abstract

**Rationale:**

Patients reporting high PD-L1 expression have shown to respond well to immunotherapy; however, some patients develop hyperprogressive disease upon initiation of immune checkpoint inhibitors. We report a patient with lung cancer and 100% PD-L1 expression who developed hyperprogressive disease while treated with pembrolizumab and responded well to salvage chemotherapy with carboplatin and pemetrexed.

**Patient concerns:**

A 66-year-old African American female with 25-pack year smoking history, diabetes mellitus type 2, essential thrombocytosis, and a history of papillary thyroid carcinoma developed relapsed lung adenocarcinoma after 13 months of no evidence of disease.

**Diagnosis:**

Surveillance imagine showed subcarinal and hilar lymphadenopathy, which was confirmed as recurrent lung adenocarcinoma via bronchoscopy. In addition, a brain scan showed a 5 mm enhancing left insular lesion. PD-L1 was reported as 100% expression. Staging was reported as stage IVB TxN3M1c lung adenocarcinoma.

**Interventions:**

One fraction of radiation with a total dose of 20 Gray was delivered to the left insular lesion. The patient initiated pembrolizumab (200 mg) every 3 weeks. She was then treated with salvage chemotherapy consisting of carboplatin (AUC 5) and pemetrexed (500 mg/m^2^) every 3 weeks for 3 cycles.

**Outcomes:**

The brain lesion resolved after the radiation therapy. The patient developed hyperprogression with a large pericardial effusion and right pleural effusion after 2 treatments of pembrolizumab. Her PD-L1 expression decreased from 100% to 0% over a 10-week period. Salvage chemotherapy with carboplatin and pemetrexed resulted with 20 months of ongoing to evidence of disease.

**Lessons:**

Immune checkpoint inhibitor-related hyperprogressive disease may respond to second-line salvage chemotherapy. Complete PD-L1 expression loss was observed after the patient's treatment and could be a marker of hyperprogressive disease or tumor immunoevasion.

## 1 Introduction

The treatment of nonsmall cell lung cancer (NSCLC) has drastically changed in the recent years and in 2015 the Federal Drug Administration (FDA) approved the first-use of immunotherapy, pembrolizumab, in NSCLC.^[1]^ This has led to the widespread adoption of immune checkpoint inhibitors (ICIs) that has yielded not only positive results in progression-free survival (PFS) but also dramatic benefit in overall survival (OS). The use of programmed death-ligand 1 (PD-L1) testing has been used as a surrogate marker with several studies showing that patients with PD-L1 proportion score greater than 50% tend to respond better to immunotherapy.^[2,3]^ A most recent study identified a 5-year OS of 29.6% for patients with PD-L1 proportion score of 50% or greater.^[4]^ Nevertheless, PD-L1 is not a completely reliable marker of response, and in some cases, patients with PD-L1 expression ≥1% still respond well to immunotherapy.^[5]^ However, a few studies have recently reported that patients with PD-L1 expression may fail to respond to immunotherapy and sometimes have immediate hyperprogressive disease (HPD) after immunotherapy treatment.^[6,7]^ Herein, we describe a case of a 66-year-old African--American woman who had 100% PD-L1 (22C3) expression and almost immediately progressed after single-agent pembrolizumab.

## 2 Case presentation

A 66-year-old African--American female with a 25-pack year smoking history, diabetes mellitus type 2, and essential thrombocytosis, initially presented with right supraclavicular lymphadenopathy. The patient had a prior history of papillary thyroid carcinoma treated with total thyroidectomy followed by radioactive iodine ablation therapy. A biopsy of the right supraclavicular neck mass was initially reported as poorly differentiated papillary carcinoma with anaplastic changes; however, it was later reported as lung adenocarcinoma. Immunohistochemistry (IHC) staining showed strong, diffuse nuclear reactivity for TTF-1 and strong cytoplasmic staining for CK7, while thyroglobulin and CK20 were negative. A positron emission tomography-computed tomography (PET-CT) revealed fluorodeoxyglucose (FDG) avid mediastinal lymphadenopathy but no primary lung lesion. An endobronchial ultrasound fine-needle biopsy of the lymph nodes suggested a poorly differentiated adenocarcinoma of lung origin with IHC staining positive for TTF-1 and Napsin-A and negative for thyroglobulin and PAX-8. The patient was staged as IIIB adenocarcinoma of the lung with unspecified laterality. Molecular evaluation of the subcarinal lymph node tissue showed no gene mutation or rearrangement in EGFR, ALK, or ROS1. She received concurrent chemoradiation to the chest and right neck mass and 2 cycles of adjuvant chemotherapy with carboplatin and paclitaxel. The treatment was tolerated well and resulted in complete response.

The patient had no evidence of disease for 13 months before a surveillance CT of the chest, abdomen, and pelvis showed subcarinal and hilar lymphadenopathy, and a subsequent PET-CT displayed FDG avidity in the lymph nodes. Bronchoscopy needle biopsy of the station 4L lymph node confirmed recurrent lung adenocarcinoma. Molecular testing on the biopsied tissue via next-generation sequencing (NGS) reported 3 mutations (ERBB3 H292Y, STK11 A218Lfs∗69, and TP53 R283P) and amplification of AKT1, MYC, NTRK1 as well as 5 variants of unknown significance: ATM P604S, ATM R832C, BRCA2 S2835P, CDKN2B D86N, and MLH1 R487Q. In addition, the PD-L1 (22C3) testing showed high expression with a tumor proportion score of 100% and an intensity of 3+. Liquid biopsy utilizing Guardant 360 detected three mutations (STK11 A218fs, TP53 R283P, RB1 R661Q), and 1 variant of unknown significance; PIK3CA M789T. A magnetic resonance imaging (MRI) of the brain discovered a 5 mm enhancing left insular lesion with surrounding vasogenic edema, likely metastasis. She completed 1 fraction of stereotactic radiosurgery (SRS) to the left insular lesion with a total radiation dose of 20 Gray (Gy). She was restaged as a stage IVB TxN3M1c lung adenocarcinoma.

**Figure 1:**
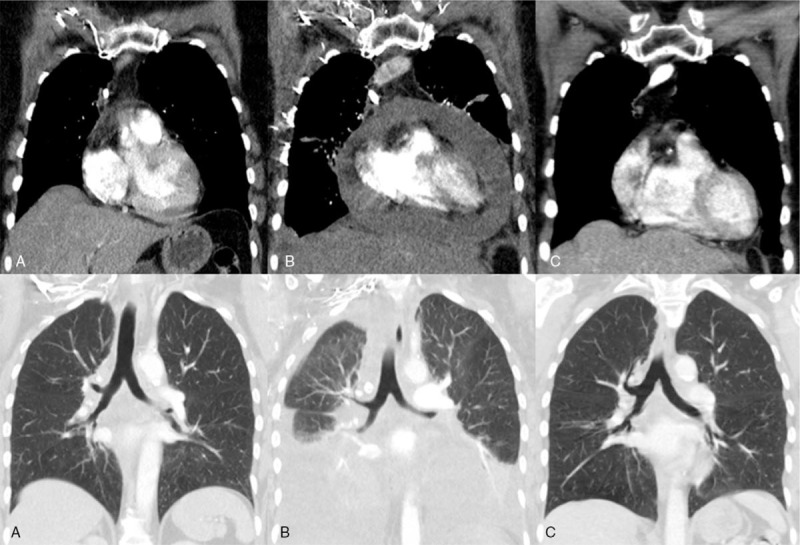
Chest CT scan of patient displaying hyperprogression top. Mediastinum view of pericardial effusion. Bottom. Lung view of pleural effusion. (A) Before pembrolizumab, (B) Hyperprogression, (C) Response to chemotherapy.

**Figure 2:**
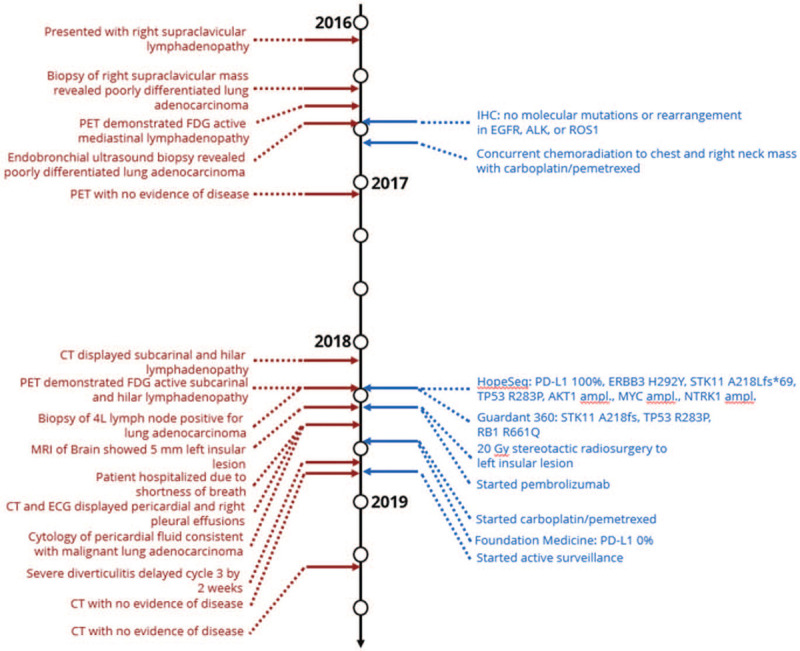
Clinical, radiographic and molecular events throughout treatment history. The left column timeline displays clinical and radiographic information while the right column timeline indicates treatment regimens and molecular testing data.

After completing brain SRS, the patient began systemic treatment with standard-dose (200 mg) single-agent pembrolizumab every 3 weeks. Minimal side effects of mild diarrhea and joint stiffness were reported after the first cycle of pembrolizumab. She developed nausea and fatigue after the second cycle and was admitted to the hospital for shortness of breath 2 weeks later. A chest CT and echocardiogram revealed a large pericardial effusion with a large right pleural effusion. An emergency pericardiocentesis and thoracentesis drained the effusions, and cytology review indicated malignancy, consistent with poorly differentiated carcinoma. The pericardial fluid was sent to Foundation Medicine and PD-L1 (22C3) testing reported a negative result with a tumor proportion score of 0%, but the remaining sample was insufficient for additional DNA testing. Thoracic tumor board reviewed the patient's case and agreed that pembrolizumab should be discontinued due to HPD (Fig. 1). Systemic therapy was switched to carboplatin (AUC 5) and pemetrexed (500 mg/m^2^) every 3 weeks. She tolerated the first 2 cycles well before developing severe diverticulitis. The third cycle was delayed 2 weeks while she completed her antibiotic course of ciprofloxacin and metronidazole. Treatment was discontinued after the third cycle in favor of active surveillance; she has had no evidence of disease over the last 20 months. Treatment history is summarized and visualized in Figure 2.

## 3 Discussion

In this case study, the patient described had 100% PD-L1 expression and developed HPD 5 weeks into her treatment plan after 2 cycles of pembrolizumab. HPD was observed in 2 new metastatic sites with emerging large pericardial and pleural effusions. There have been a number of retrospective studies analyzing HPD in NSCLC.^[8–11]^ A few of these studies defined HPD only via radiological evaluation of tumor growth kinetics and tumor growth rate in the target lesions according to RECIST version 1.1.^[8,9]^ Although this technique would capture the majority of patients, it would not in our case, as the patient had no primary lung lesion to observe. Clinical criteria including PFS <2 months alongside radiological criteria were used in other studies to evaluate these patients.^[10,11]^ The need to establish both universal clinical and radiological definitions of HPD is imperative moving forward.

After disease recurrence, a biopsy was conducted on one of the FDG avid mediastinal lymph nodes, and PD-L1 (22C3) testing showed high expression with a tumor proportion score of 100%. However, the pericardial fluid from the emergency pericardiocentesis was PD-L1 (22C3) negative. Tumor cell characteristics changed from 100% PD-L1 high to 0% PD-L1 after only two cycles of single-agent pembrolizumab. Reliability of PD-L1 IHC testing on pleural/pericardial fluid has been shown between matched PD-L1 histological specimens and pleural cytology blocks with significant correlation and concordance.^[12]^ Two autopsy case reports have documented the loss of PD-L1 expression in 3 patients with NSCLC after exposure to immunotherapy.^[13,14]^ In 2 patients, PD-L1 expression decreased from 75.6% to 13.2% and from 100% to 58.8%.^[13]^ A third patient PD-L1 expression reduced from 98% to <1%.^[14]^ The loss of PD-L1 expression may be one mechanism by which NSCLC and other cancers treated with ICIs evolve resistance to immunotherapies. Perhaps the rate at which PD-L1 is lost could be an indicator of HPD. Future investigation of the mechanisms of PD-L1 loss is warranted in cases with HPD and immunotherapy treatment failure.

Importantly, after the patient discontinued pembrolizumab due to the HPD, she recuperated enough to receive second-line systemic chemotherapy with carboplatin and pemetrexed. Despite developing severe diverticulitis during the course of her chemotherapy treatments, she successfully completed 3 cycles of carboplatin and pemetrexed. Patients who were treated with salvage chemotherapy after immunotherapy have shown promising overall response rate (ORR).^[15,16]^ Although large cohort studies need to be performed to validate the response of salvage chemotherapy, our patient is currently off treatment and has been on active surveillance with no evidence of disease for 20 months.

NGS and liquid tumor biopsies both revealed both STK11 and TP53 mutations. STK11/TP53 comutations are associated with longer OS than other STK11 comutations.^[17]^ Recently, a study demonstrated a link between STK11 and HPD in NSCLC with (3/16) HPD patients harboring an STK11 mutation versus (0/28) without HPD.^[9]^ Although the 3 patients had KRAS comutated tumors, another study reported STK11 mutated tumors have significantly lower ORR to ICIs versus nonmutated STK11 tumors [0% (0/11) vs 34.5% (19/55)] and shorter median PFS (1.7 vs 19.3 months) and OS (11.1 vs 26.5 months).^[18]^ In a larger cohort looking at 377 patients with metastatic nonsquamous NSCLC treated with pembrolizumab, platinum-doublet chemotherapy (carboplatin or cisplatin), and pemeterexed, 102 patients harboring an STK11 genomic alteration were associated with significant differences in ORR (32.6% vs 44.7%), and significantly shorter mPFS (4.8 vs 7.2 months) and mOS (10.6 vs 16.7 months).^[19]^ STK11 and KEAP1 comutations occur in approximately 13% of all lung cancers with a higher median tumor mutational burden (9.4 vs 6.1) and are associated diminished outcomes in PFS and OS when treated with ICIs.^[20]^ STK11-mutated patients represent a subgroup of NSCLC patients where new treatment strategies are needed. Continued evaluation of STK11 mutations and their role in HPD in NSCLC is reasonable.

## 4 Conclusion

The application of immunotherapy in NSCLC has transformed lung cancer treatment; however, there is still no true biomarker to predict response or resistance to immunotherapy. Previous findings have associated a longer duration of response in patients with high PD-L1 expression. However, here, we presented a case of a patient with lung adenocarcinoma and 100% PD-L1 expression who did not respond to immunotherapy and developed immediate progression. Furthermore, the subsequent tissue showed complete loss of PD-L1 expression following immunotherapy treatment before the initiation of chemotherapy. The patient responded to chemotherapy and continues with no evidence of disease. This suggests that further evaluation of PD-L1 expression and other biomarkers such as STK11 may help us to understand immunotherapy hyperprogression and therapeutic options for this subgroup of patients.

## 5 Consent

The City of Hope Institutional Review Board approved of the research in this case study. The patient provided informed consent for publication through City of Hope's IRB# 07047.

## Acknowledgments

The authors would like to thank City of Hope nurses and supportive staff for their dedication to their patients.

## Author contributions

**Conceptualization and design:** Jeremy Fricke, Isa Mambetsariev, Rebecca Pharaon, Ravi Salgia.

**Data curation:** Jeremy Fricke, Isa Mambetsariev, Rebecca Pharaon.

**Methodology:** Shanmuga Subbiah, Swapnil Rajurkar.

**Investigation:** Shanmuga Subbiah, Swapnil Rajurkar.

**Supervision:** Ravi Salgia.

**Writing – original draft:** Jeremy Fricke.

**Writing – review & editing:** All authors contributed to the review, editing, and approval of the final manuscript.
